# Sex steroid priming in short stature children unresponsive to GH stimulation tests: Why, who, when and how

**DOI:** 10.3389/fendo.2022.1072271

**Published:** 2022-11-29

**Authors:** Cristina Partenope, Elena Galazzi, Assunta Albanese, Simonetta Bellone, Ivana Rabbone, Luca Persani

**Affiliations:** ^1^ Division of Pediatrics, Department of Health Science University of Piemonte Orientale, Ospedale Maggiore della Carità, Novara, Italy; ^2^ Department of Endocrinology and Metabolic Diseases, Istituto Auxologico Italiano IRCCS, Milan, Italy; ^3^ Department of Paediatric Endocrinology, St. George’s University Hospital NHS Foundation Trust, London, United Kingdom; ^4^ Department of Medical Biotechnology and Translational Medicine, University of Milan, Milan, Italy

**Keywords:** pubertal delay, sex steroid priming, GH deficiency (GHD), short stature, peripubertal age, growth hormone stimulation test (GHST)

## Abstract

Despite decades of experience, the diagnosis of growth hormone deficiency (GHD) remains challenging, especially in peripubertal children. Failure to respond to GH stimulation tests (GHSTs) is needed to confirm GHD, but long-standing controversies regarding the number of tests needed and the interpretation of GH peaks are still a matter of debate worldwide. Diagnostic workup is even more problematic in short children with slow growth and delayed sexual development: they often exhibit low GH peaks under GHST, which often normalize as puberty progresses. Consequently, this transient suboptimal response to GHST may result in GH overtreatment, carrying both health and economic concerns. Considering the complex and bound link between GH axis and sex steroids, the use of sex steroid priming prior to GHST might be helpful in peripubertal setting. However, its use is still controversial. There is no consensus regarding patient selection, timing, dose, and preparation of sex steroids. In this review, we aim to overview the use of sex steroid priming in clinical practice, highlighting the need to develop appropriate guidelines in order to overcome diagnostic pitfalls in peripubertal age.

## Introduction

Despite decades of experience, the diagnosis of growth hormone deficiency (GHD) remains a challenge for the paediatric endocrinologist. It should result from “auxologic, anatomical and laboratory data”, as stated in the recent document from Growth Hormone Research Society Workshop (1), and therefore appropriate selection of patients eligible for growth hormone (GH) investigation is crucial. Family and previous medical history should be taken into account, as well as accurate physical examination to rule out body disproportions and syndromic features and to evaluate pubertal status. Radiological findings such as brain MRI for hypothalamus-pituitary study and hand-wrist X-ray for bone age assessment do also contribute to the diagnostic evaluation. Finally, serum IGF-I and IGFBP 3 values are supportive biochemical findings. Since measurement of random serum GH concentrations are useless, except for neonates (2), failure to respond to GH stimulation tests (GHSTs) is needed to confirm GHD, when an alternative aetiology for short stature is not evident.

Long-standing controversies continue to generate debate, regarding how to perform and interpret GHSTs (3–7). Arbitrary and not universally adopted cut-off levels, reliability and reproducibility of these tests are the main issues. In a study by Marin et al. investigating GH response to provocative tests in prepubertal children with normal stature, 61% of them failed three different tests with a cut-off fixed at 7 mcg/l (8). Difficulties in distinguishing partial GHD from idiopathic short stature (ISS) or constitutional delay of growth and puberty (CDGP) have already been extensively highlighted (9–12), showing normalization of GH peaks at early retesting (10). Both GH and sex steroids are required for the pubertal growth spurt and there is strong evidence, at least in boys, that sex steroids are a potent stimulus facilitating GH release (13). Diagnostic workup is challenging in short children with slow growth velocity and delayed sexual development: they often exhibit low GH peaks under GHST, which reverts to normal levels as puberty progresses. As a consequence, this transient suboptimal response to GHST may result in GH overtreatment, with both health and economic concerns (4).

According to the 2019 GH Research Society guidelines, the use of sex steroid priming prior to GHST might be helpful in the peripubertal setting. It was first introduced in 1968 to reduce the percentage of false positive results to GHST, since availability of GH treatment was limited at that time (14). However, its use is still controversial. There is no consensus regarding patient selection, timing, dosage, preparation, and administration of sex steroids.

In this review, we aim to overview the use of sex steroid priming in clinical practice, highlighting the need to develop appropriate guidelines in order to overcome diagnostic pitfalls in peripubertal age.

## Rationale for use of priming

After minipuberty occurs, during childhood the hypothalamus-pituitary-gonadal system becomes quiescent. A significant change and maturation in the hypothalamic “gonadostat” occur in girls at approximately 10.5 years and in boys at 11.5 years. GnRH neurons amplify their signal to increase amplitude and frequency of FSH and LH pulsatile release by the pituitary gonadotropic cells with a prominent nocturnal rhythm (15). This in turn triggers sex steroid production by gonads with feedback regulation of gonadotropin secretion by both testosterone and oestrogen (16). In girls growth acceleration starts with the onset of breast development (Tanner Stage B2), whereas in boys the rate of growth increases significantly only after achieving Tanner stage III-IV (with approximately 10 ml of testicular volume) (17, 18). The different timing of puberty onset between sexes may be related to an increased sensitivity of the gonadotrophs to GnRH in girls or to a greater bioactivity of oestrogen in prepubertal females compared to prepubertal males (19).

Historical data have demonstrated a complex and close link between GH axis and sex steroids both in animals models and humans (20–24). The hypothalamic regulation of GH secretion results primarily from a stimulating control by GH-releasing hormone (GHRH) and by an inhibiting control by somatostatin. On one hand, sex steroids are known to potentiate GH responsiveness to GHRH in somatotroph cells in the anterior pituitary gland; on the other hand, GH modulates pubertal development by stimulating local production of insulin-like growth factors in gonads and by enhancing gonadal response to gonadotrophin secretion and these axes constitute a regulated network whose feedback relationships manifest important changes at the time of puberty.

The use of sex steroid priming in the diagnosis of GHD is based on three considerations:

A) GH levels increase physiologically during puberty.

Rose et al. (25) analysed circadian GH secretion of 132 normal children and adolescents (every 20 minutes for 24 hours) and found that spontaneous GH secretion increases during puberty, with a peak during early-mid puberty in girls (sometimes before the earliest clinical signs of puberty) and during mid-late puberty in boys, corresponding to their peak of growth velocity. If correlated with bone maturation, mean GH levels and pulse amplitude increased in girls beyond a bone age of 8 years, whereas a decrease in growth velocity was observed in boys till bone age of 11 years. This means that the interpretation of GH levels according to chronological age may be misleading and generate a high amount of false GHD diagnosis.

Similarly, Mauras et al. (26) confirmed that prepubertal boys showed lower GH concentrations compared to sexually mature boys of same age and these findings were secondary to variations in amplitude rather than in the pulse frequency of GH secretions. A study from a cross-sectional group of healthy North American males showed that mean 24-hour GH concentration of young adult is similar to that in the prepubertal state, suggesting that the relative impact of sex steroids on GH concentrations is limited to the last stages of puberty (27).

The role of IGF-I as a modulator of pubertal timing is increasingly recognized (28, 29). High GH secretion is most certainly responsible for the increased IGF-I levels during puberty; nevertheless, previous studies have found a suboptimal growth response to GH stimulation test in girls with central precocious puberty (30, 31). Negative feedback of IGF-I levels on pituitary may be reduced in puberty, emphasizing their synergic anabolic role during growth spurt. IGF-I levels peak 2 years after growth spurt and might play a role in gonadal and secondary sexual characteristics maturation (32).

B) Sex steroids regulate GH secretion and actions, both directly or *via* modulators, through paracrine or endocrine signalling (33).

The evidence of high levels of oestrogen receptors in hypothalamus and pituitary demonstrates that oestrogens act as regulator of GH secretion by reducing somatostatin receptor expression, increasing the number of GHRH-binding sites and increasing ghrelin-induced GH production (34). Moreover, 80% of the somatotropes in human pituitary co-express aromatase, and in patients with aromatase deficiency the GH response to stimulation is substantially blunted (35). Similarly, late pubertal boys receiving oestrogen receptor blocker (Tamoxifen) to evaluate the role of endogenous oestrogens in the control of GH secretion showed a significant decrease in GH production rates, in mean GH pulse amplitudes, and in serum IGF-I levels (36). These data support the paracrine effect of oestrogens derived from aromatization of androgens in men. Peripherally, oestrogen exerts tissue-specific effect: for example, in bones it potentiates GH signalling *via* SOCS-2 pathway promoting osteoblast proliferation and bone growth (37). Testosterone also acts peripherally, amplifying GH-mediated secretion of IGF-I, sodium retention, substrate metabolism and protein anabolism, while exhibiting similar but independent actions of its own and interacts directly with GH in the liver to regulate protein metabolism by enhancing GH receptor expression (38). Contrary to androgens, oestrogens do not influence whole body protein anabolism, and this may explain sex differences in muscle bulk. Sex steroids modulate GH secretion during lifespan. Evidence of a regulatory role of sex steroids on GH comes from association studies in children and adults. Physiologically, in children, a positive correlation between sex steroid and GH status has been proved from the evidence of a threefold increase in GH secretion along with an increase in gonadal steroid concentrations during puberty (39).

C) Exogenous sex steroids stimulate GH synthesis, release, and action.

Sex steroids administration exerts a stimulatory effect on GH secretory episodes. In the above mentioned study by Marin et al. 40 mcg/m2 oral ethynil oestradiol given two days before GHST increased GH peak in normal prepubertal children from 1.9-20 mcg/L to 7.2-40.5 mcg/L, reaching similar levels of pubertal children (8-63.2 mcg/L) (8). Low doses of ethinyloestradiol (0.1 mcg/kg/day) could rise GH concentrations after 1 to 5 weeks and improve height gain in patients with Turner Syndrome, without significantly advancing bone maturation (40). The effect of oestrogen on GH secretion is dependent on the route of administration. When administered orally, oestrogen reduces hepatic IGF-I production as a result of first-pass effect. The fall in IGF-I after oral oestrogen therapy reduces negative feedback on GH secretion, as seen in postmenopausal women (41). In men with hypogonadism, testosterone replacement stimulates GH/IGF-I system peripherally and enhances tissue responsiveness to GH. Importantly, non-aromatizable androgens do not stimulate GH secretion (42).

## Concerns and benefits

A recent audit among nine American and European expert paediatric endocrinologists showed that priming is recommended in 5 out of 9 countries (the UK, the Netherlands, Denmark, Spain, and Germany), but protocols differ significantly (43). The prevalence was higher (up to 85%) among tertiary endocrine centres in UK (44). In contrast, different data result from a French population-based registry (45): in 2,165 patients with idiopathic GHD sex steroid priming was used in only 2% of patients before GHST. Pubertal development has been reported not to increase GH reserve when evaluated by GHST (3, 46). It should be noted, however, that these studies were performed decades ago with different GH assays and sometimes with obsolete and unreliable diagnostic test (i.e. treadmill exercise). Soliman et al. found that the mean GH response to provocative testing did not differ between primed and not primed-children, although testosterone intramuscular injections were administered at a lower dose compared to other reports; this study however included younger children (starting from 9 years old) compared to other papers and this could had influenced the results (47). Another concern against routine use of priming is that primed GH peak may be unphysiological and transient, therefore many peripubertal GH deficient patients may be not identified, preventing them from receiving appropriate and potentially beneficial treatment (13, 48, 49). The existence of “transient GHD” in adolescents with delayed puberty is still debated, as the underlying pathology is more often consistent with sex hormones deficiency rather than GHD. The majority of the patients with idiopathic GHD show normal GH secretion when retested after achieving of final height, whereas the likelihood of permanent GHD is higher in adults with congenital panhypopituitarism and acquired pituitary lesions (50, 51). For this reason some authors suggested the need to retest patients with idiopathic GHD after one year of therapy: the Belgian Study Group for Paediatric Endocrinology reported normal GH peak in 44% of cases (52). 28 out of 33 GHD patients of an Italian cohort with normal pituitary morphology at brain MRI normalized GH secretion even before commencement of GH treatment (10). Recently published data suggested patients with isolated GHD without a hypothalamic-pituitary abnormality on MR scanning (including small anterior pituitary) can also be considered for early retesting of the GH axis once they are established in puberty (Tanner stages B2/3 in girls & 6-12 ml testes in boys) (53). In addition, GH treatment seems to have little effect on final height in adolescents with transient GHD (54).

On the contrary, many other studies reported that sex steroid priming could improve diagnostic efficacy of GHSTs in peripubertal patients. Molina et al. demonstrated that 53.8% of short children who underwent clonidine stimulation test normalized GH secretion after priming (55); these data were confirmed also among children affected by ISS compared to GHD when micronized oestradiol was administered before GHST (56). A prospective study including 50 boys with poor growth who failed to respond to unprimed GHST showed that some of them normalized GH response to GHST after testosterone priming with different protocols (57): 31/50 boys after single low dose testosterone (62.5 mg/m2), 11/50 after single conventional dose (125 mg/m2) and 8/50 boys after multiple-dose testosterone (62.5 mg/m2 weekly for 4 weeks). Mean peak GH increased from 4.9 ± 3.0 to 19.3 ± 5.9 mcg/L in the low dose group, from 5.4 ± 2.1 to 17.0 ± 5.9 mcg/L in the conventional dose group, and from 5.1 ± 2.1 to 15.4 ± 5.1 mcg/L in the multiple-dose group. There was no statistical difference among mean peak GH level of the three groups before and after priming. Most relevantly, those subjects were able to reach a final height well within their genetic target (mean final height -1.27 +/-0.72 SDS versus mean mid-parental height -1.38 +/-0.72 SDS) without any rhGH treatment. More recently, a retrospective study among ENDO-ERN centres confirmed that sex steroid priming enhanced the specificity of GHST in differential diagnosis between GHD and CDGP in a cohort of 184 peripubertal children (74 females), selecting children who may benefit the most from priming. In fact, those children diagnosed as GHD upon a primed GHST reached a greater final height compared to untreated CDGP (primed CDGP vs GHD FH: -1.5 vs -0.81; p = 0.023) and closer to their midparental target (primed CDGP vs GHD ΔSDS FH-TH: -0.74 vs 0.12, p = 0.025), whereas those diagnosed upon an unprimed GHST, final height was similar between GHD children treated with rhGH and untreated CDGP children (unprimed CDGP vs GHD FH: -0.9 vs -0.93, p =n.s.)

(58). Lastly, two recent retrospective Italian studies on short pre/peripubertal boys primed with a prolonged low-dose testosterone protocols (either with intramuscular or transdermal preparation) showed an increase in height and growth velocity SDS and a normalization of GHST peaks compared to untreated boys (59, 60).

A summary of the clinical research studies included in this review is reported in **Table 1**.

**Table 1:** Summary of the main clinical studies included in the review.

STUDY	STUDY TYPE	COHORT SIZE	SEX STEROID	FINDINGS
Marin et al., 1994 (8)	Randomized control trial	84	Both boys and girls: EE 40 mcg/m^2^ divided into 3 doses For 2 days prior to GHST	Priming increased GH response to GHST in pubertal and prepubertal children with normal height
Drop et al., 1982 (13)	Control study	8	Boys: TU 120 mg twice a day For 5 days prior to GHST Girls: EE 50 mcg twice a day For 5 days prior to GHST	Priming (unlike spontaneous puberty) did not increase GH response to GHST in GHD children
Soliman et al., 2014 (47)	Randomized control trial	92	Boys: TD 25 mg 7-10 days before GHST Girls: Conjugated oestrogens 1.25 mg daily For 3 days prior to GHST	Priming did not increase GH response to GHST in prepubertal children (age 9-13 years)
Chalew et al., 1988 (48)	Control study	8, all boys	TD 200 mg once a month for 4-5 months before assessment of spontaneous GH secretion	Testosterone transiently increases spontaneous GH secretion in boys with CDGP
Molina et al., 2008 (55)	Control study	39	Boys: TD 100 mg 5-8 days prior to GHST Girls: EV 1-2 mg daily For 3 days prior to GHST	Priming normalized GH response to GHST in both GHD and CDGP
Martinez et al., 2000 (56)	Randomized control trial	59	Both boys and girls: EV 1-2 mg daily For 3 days prior to GHST	Priming increases GH response to GHST in both GHD and ISS children
Gonc et al., 2008 (57)	Retrospective cohort study	50, all boys	Testosterone Esters (Sustanon^®^) 62.5 or 125 mg/m^2^ 7 days before GHST In non-responders (stimulated GH peak <10 ng/ml): multiple-dose priming (62.5 mg/m^2^ monthly injections for 3-4 months)	Priming normalized GH response to GHST in 42/50 peripubertal boys. 8/50 elicit normal GH peaks after multiple dose priming. All 50 patients were able to achieve a final height within their mid-parental target, regardless of priming regimen.
Galazzi et al., 2020 (58)	Retrospective cohort study	184	Mixed regimes: Boys: TD - Low dose: 50mg - High dose: 100mg 4-7 days before GHST Girls: EE 100 mcg daily For 3 days prior to GHST or Stilboestrol 1 mg twice a day For 2 days prior to GHST	Priming played a key role in identifying children who may benefit most from recombinant GH treatment in terms of final height. Priming enhances the diagnostic accuracy of GHST in the differential diagnosis between peripubertal GHD and CDGP.
Chioma et al., 2018 (59)	Retrospective study	73, all boys	- TD 50 mg every 4 weeks for 3 months - Transdermal testosterone 2% 10 mg daily for 3 months	Both testosterone preparations (intramuscular and transdermal) were able to increase SDS height and SDS growth velocity compared to placebo, helping in the differential diagnosis between peripubertal GHD and CDGP.
Mastromattei et al., 2022 (60)	Retrospective study	246, all boys	- TD 50 mg every 4 weeks for 3 months - Transdermal testosterone 2% 10 mg daily for 3 months	3-month low dose priming with testosterone (either intramuscular or transdermal) increased height and growth velocity and normalized almost all GHST and IGF-1 levels in short pre/peripubertal boys. Testicular enlargement and LH increase was more evident with transdermal testosterone preparations.
Moll et al., 1986 (61)	Control study	23	Both boys and girls: EE 20 mcg/m^2^ daily For 1-2 days prior to GHST	Higher priming EE regimen increased GH response to GHST in prepubertal children (age 3-15 years)
Borghi et al., 2006 (62)	Control study	22	Both boys and girls: Transdermal EE 50 mcg For 3 days prior to GHST	Priming increased GH response to GHST in non-GHD short children
Bacon et al., 1969 (63)	Control study	26	Both boys and girls: Stilboestrol 5 mg twice a day For 3 days prior to GHST	Oestrogen increased GH secretion similarly to arginine test
Ross et al., 1987 (64)	Control study	14	Both boys and girls Stilboestrol 1 mg twice a day For 2 days prior to GHST	Priming increased GH response to GHST through the hypothalamus presumably by increasing endogenous GHRH release
Lanes et al., 1986 (65)	Control study	144	Both boys and girls: Conjugated oestrogens 1.25 mg daily For 3 days prior to GHST	Priming did not seem to alter the GH response to exercise in prepubertal children
Wilson et al., 1993 (66)	Control study	73	Both boys and girls: Conjugated oestrogens 2.5 mg 1 dose the evening before and 1 dose the morning of the GHST	Priming did not increase GH response to GHST (clonidine)
Gonc et al., 2001 (67)	Control study	84, all boys	Testosterone Esters (Sustanon^®^) 62.5 or 125 mg/m^2^ 7 days before GHST Or 62.5 mg/m^2^ monthly (three doses in total) last dose 7 days before GHST)	Priming either with low or high dose increased GH response to GHST in peripubertal boys. Multiple-dose priming is useful in those patients who failed to respond to a GHST after a single-dose priming

EE, Ethinyloestradiol; TU, Testosterone undecanoate; TD, Testosterone depot; TP, Testosterone Propionate; EV, oestradiol valerate.

## Use of priming in clinical practice

The actual limitation for use of priming is the current absence of standardized protocol for sex steroids administration with reference to patients’ age, type, dose, and timing. The 2019 international audit (43) revealed that priming may be used in boys between the ages of 10 to 13 years and in girls between the ages of 8 to 12 years. According to 2016 Pediatric Endocrine Society Guidelines priming should be considered in prepubertal boys older than 11 and in prepubertal girls older than 10 years with final height prognosis > –2 SD of the reference population (68). Another adopted strategy is to prime all prepubertal children (boys >9 years, girls >8 years, either on chronological age or bone age) (4). Lazar and Phillip (49) advocated the use of priming only in selected cases, i.e. girls aged > 11.5–12 years and boys aged > 13–13.5 years with any or only initial signs of puberty. Similarly, the recent update from GH Research Society advised to limit its use to adolescents with delayed puberty only, but did not provide any age cut-off due to lack of consensus (1).

Published data revealed a large heterogeneity in current practice about dose and type of sex steroid preparations across centres and countries, as listed in **Tables 2**, **3**.

**Table 2:** Sex steroid priming regimens for girls.

	Dose	Route of administration	Timing prior to GHST
β-oestradiol (oestradiol valerate)	1 mg < 20 kg daily (bedtime) 2 mg >20 kg (55, 56, 68)	Oral	For 2 (58) – 3 (55, 56) days
Ethinyloestradiol	10 or 20 or 30 or 50 mcg (69) 50 mcg twice a day (13) 100 mcg (58, 70) 20 mcg/m2 daily (bedtime) (61) 40 mcg/m2 daily (8) 50 mcg patch (62)	Oral Oral Oral Oral Oral Oral Transdermal	For 2 or 3 or 5 or 7 days For 5 days For 3 days For 1-2 days For 2 days For 2 days 1 patch to be kept on for 3 days
Stilboestrol	5 mg twice a day (63) 1 mg twice a day (58, 64)	Oral Oral	For 3 days For 2 days
Premarin^®^ (Conjugated oestrogen)	1.25 daily (47, 65) 2.5 mg (66)	Oral Oral	For 3 days 1 dose the evening before and 1 dose the morning of the GHST

**Table 3:** Sex steroid priming regimens for boys.

	Dose	Route of administration	Timing prior to GHST
Testosterone depot (Testosterone Enanthate)	25 mg (47) 50 – 100 mg (58, 68) 100 mg (4, 55)	Intramuscular	7-10 days 7 days 5-8 days (55), 7-10 days (4)
Sustanon^®^ (mixture of Testosterone esters: propionate, phenylpropionate, isocaproate, decanoate)	50-100 mg (69) 62.5 mg/m2 (57, 67) 125 mg/m2 (57, 67) MDT (multiple dose testosterone) 62.5 mg/m2 monthly for 3 doses (67)	Intramuscular	3-5 days 7 days 7 days Last injection 7 days before test
Testosterone undecanoate	120 mg daily (13)	Oral	5 days
Testosterone gel 2%	10 mg daily for 6 months (59, 60)	Transdermal	3-6 months before test
β-oestradiol (oestradiol valerate)	1 mg < 20 kg 2 mg >20 kg daily (bedtime) (52, 56)	Oral	For 2 (68) – 3 (52) days
Ethinyloestradiol	10 or 20 or 50 or 100 mcg (69) 20 mcg/m2 daily (bedtime) (61) 40 mcg/m2 daily (8) 50 mcg patch (62)	Oral Oral Oral Transdermal	For 3 days For 1-2 days For 2 days 1 patch to be applied and kept on for 3 days
Stilboestrol	5 mg twice a day (63) 1 mg twice a day (64)	Oral Oral	For 3 days For 2 days
Premarin^®^ (Conjugated oestrogen)	1.25 daily (61, 65) 2.5 mg (66)	Oral Oral	For 3 days 1 dose the evening before and 1 dose the morning of the GHST

A reasonable, easy to use and commonly used approach in both boys and girls would be 2 mg (1 mg for body weight <20 kg) of β-oestradiol orally on each of the 3 evenings preceding the test, as indicated in the BSPED UK Consensus National Guidelines for Sex Hormone Priming (71). Alternatively, for instance in case of lack of supply of β-oestradiol, children of both sexes can be primed with oral Stilboestrol (1 mg twice a day for 2 days before the test). Promising results have been proven for boys primed with transdermal testosterone 2% (59, 60).

As previously mentioned, clinical and experimental data strongly suggest that oestrogens control the feedback amplification of GH levels during puberty even in males and that the modulation of GH production by androgens is mainly secondary to their aromatization. Moreover, the use of oral or transdermal preparations is suitable for needle-phobic patients and could possibly increase patient compliance to treatment. Several oestrogen and testosterone products are available and their effects and pharmacokinetics may vary according to different route and strength, so that a comparison is not always possible (72, 73). For example, oral ethynyloestradiol elicits a sharp response on IGF-I and achieves its peak plasma concentration within 0.5-1.5 hours with a half-life of approximately 12-14 hours, whilst transdermal formulations result in lower oestrogen metabolite concentrations. In a pilot study by Borghi et al. oestrogen patches are considered safe and viable since they deliver a continuous release of oestradiol, guaranteeing stable plasma levels for 72 hours (70). In addition, this route of administration avoids the first passage hepatic effect and does not directly affect IGF-I synthesis. Testosterone transdermal gel are commonly accepted for puberty induction in boys with CDGP and hypopituitarism (74) and their use can be theorized for priming prior to GHST as suggested in the study by Mastromattei et al. 2022 (60).

For research purposes, Radetti et al. reported that priming GHST with Pegvisomant, a GH receptor antagonist, would enhance the accuracy of the test, although this approach has not been extensively confirmed in clinical practice (75).

## Side effects

Data are lacking on potential side effects of sex steroid priming. Albrecht et al. analysed the consequences of priming with testosterone enanthate i.m. (50 mg, 125 mg, 250 mg) given 7 days before GHST on 188 prepubertal boys. Overall, only 5 subjects displayed side effects (2.7%), irrespective of testosterone plasma levels: 2/188 developed severe priapism requiring cavernosal aspiration (after testosterone 125 mg single dose), 1/188 mild self-limiting priapism, 2/188 complained testicular pain (after testosterone 50 mg single dose (76). Other common adverse effects related to intramuscular administration are local inflammation and pain at injection site. It is worth mentioning that, as cottonseed or sesame/peanut oil are the formulation vehicle, testosterone vials are contraindicated in case of known hypersensitivity/allergy to nuts or soy (74). Side effects such as severe nausea and vomiting are frequently observed following priming with Stilboestrol. Transient breast tenderness has been reported as well (49).

## The need for a structured approach

Since GH production and release are significantly and physiologically influenced by androgen and oestrogen milieu during puberty, sex steroid priming has been proposed and proved to improve diagnostic performance of GH provocation tests. We therefore recommend including priming to all protocols for diagnostic workup of short patients. However, there is no consensus on who, when and how to use it. Nowadays, the availability of biosynthetic GH has eased limitations for GH prescription and has led to the conclusion that sex hormone priming is not necessary in the routine evaluation of every prepubertal child. It should be considered only in a subgroup of adolescents with delayed puberty (e.g. Tanner stages 1 and 2 in girls older than 12 years and boys older than 13 years) in order to prevent unnecessary GH treatment of children with CDGP. Assessment of bone age is warranted to evaluate pubertal delay and to select candidates for sex steroid priming. Several protocols have been suggested for priming and no one demonstrated evident superiority over others. Nevertheless, among different preparations and dosages, oral oestrogen seems preferable in both girls and boys as oestrogen plays a pivotal role in the regulation of GH secretion also in males.

## Conclusion

Although sex hormone priming prior to GHST is not mandatory to diagnose GHD, several evidence recommend its use in peripubertal children in order to select those who may benefit the most from rhGH treatment, avoiding redundant treatment in CDGP, who can either achieve normalization of their auxological parameters with a low dose short course of sex steroids. Large prospective studies following patients until final height are still needed to clarify the optimal priming regimen and the correct timing of these preparations during their growth, especially in girls.

## Author contributions

CP drafted the manuscript. AA and EG critically revised the manuscript. LP supervised the whole process. All authors contributed to the article and approved the submitted version.

## Funding

This work was partially funded by the Italian Ministry of Health, Rome (grant code:05C202_2012).

## Conflict of interest

The authors declare that the research was conducted in the absence of any commercial or financial relationships that could be construed as a potential conflict of interest.

## Publisher’s note

All claims expressed in this article are solely those of the authors and do not necessarily represent those of their affiliated organizations, or those of the publisher, the editors and the reviewers. Any product that may be evaluated in this article, or claim that may be made by its manufacturer, is not guaranteed or endorsed by the publisher.
